# Success and complication rate of miniscrew assisted non-surgical palatal expansion in adults - a consecutive study using a novel force-controlled polycyclic activation protocol

**DOI:** 10.1186/s13005-021-00301-2

**Published:** 2021-12-11

**Authors:** Heinz Winsauer, Andre Walter, Christos Katsaros, Oliver Ploder

**Affiliations:** 1 Orthodontic Office, Belruptstrasse, Bregenz, Austria; 2 Department of Orthodontics, Universitat International de Catalunya (UIC), Barcelona, Spain; 3 Department of Orthodontics and Dentofacial Orthopedics, University of Bern, Bern, Switzerland; 4 Private Office for Oral and Maxillofacial Surgery, Facesurgery.at, Feldkirch, Austria

**Keywords:** Maxillary expansion, Bone-borne, Miniscrew, Non-surgical, Adult patients, MAPE, MARPE, Success, Complication

## Abstract

**Introduction:**

Bone-borne miniscrew assisted palatal expansion (MAPE) is a common technique to improve maxillary transverse deficiency in young adolescents. Adult patients usually present a challenge, as they often require additional surgical assisted maxillary expansion (SARPE). There is still no clear statement about non-surgical expansion in adult patients using this technique. The aim of this study was to evaluate the success and complication rate of non-surgical palatal expansion in adults utilizing MAPE with a novel force-controlled polycyclic expansion protocol (FCPC).

**Methods:**

This consecutive study consisted of 33 adult patients with an average age of 29.1 ± 10.2 years (min. 18 years, max. 58 years), including one dropout patient. First, four miniscrews were inserted and after 12-weeks latency, the expander was placed and the FCPC protocol was applied (MAPE group). In case of missing expansion, a SARPE was performed (SARPE group). After maximum expansion, a cone beam CT was made and widening of the midpalatal suture was measured. The outcome variables were successful non-surgical expansion and, with sample size power above 80%, the odds of failed non-surgical expansion and associated complications were evaluated. The primary predictor variable was age. Statistical analysis was performed using R (Version 3.1) to calculate power, to construct various models for measuring the odds of requiring surgical intervention/complications, and others.

**Results:**

Successful non-surgical expansion was achieved in 27 patients (84.4%), ranging from 18 to 49 years. Mean age differed significantly between both groups (26.8 ± 8.2 years vs. 41.3 ± 9.9 years; p < 0.001). Mean expansion at the anterior and posterior palate for the MAPE group was 5.4 ± 1.5 mm and 2.5 ± 1.1 mm, respectively. Among these subjects’ complications were observed in 18.5%. Age significantly increased the odds of complications (*p* = 0.019).

**Conclusions:**

1. The success rate of MAPE among individuals aged 18 to 49 years was 84.4%.

2. A V-shaped expansion pattern in the antero-posterior dimension was mostly observed.

3. Complications were significantly associated with age.

4. A careful expansion protocol seems to be beneficial to prevent unfavorable results in adult patients.

**Trial registration:**

Consecutive cohort study, Review Board No. EK-2-2014/0016.

## Background

Widening of the maxilla in children and young adolescents is usually done with hyrax or Haas-type tooth-borne expanders. However, slow maxillary expansion (SME) of the dental arch with RPE-type appliances (rapid palatal expansion) did not produce stable increase in upper inter-canine width; this was significantly greater in the RPE group then in the SME group [1]. Due to the increased skeletal resistance, rapid palatal expansion might be recommended for patients at the final stage of pubertal growth. For adults, however, RPE has been considered rarely successful and can produce undesirable effects on the dentoalveolar complex [2, 3]. With the beginning of fusion of the midpalatal suture, maxillary widening can be treated with surgically-assisted rapid palatal expansion (SARPE) using various appliances [3, 4]. However, problems persist with respect to the need for a surgical intervention, including osteotomies, and the risk of root damage or infections, asymmetric maxillary expansion, and device-related technical problems [5–7]. Along with the development of digital techniques and bone-borne anchorage, a miniscrew or implant assisted rapid palatal expansion (MARPE) has become available for the treatment of maxillary transverse deficiency in adults [8–12]. In adults it represents a treatment solution that can potentially reduce the complications of SARPE and is minimally invasive, secure, and reliably stable [12]. However, when MARPE is used with a rapid continuously opening expansion protocol in adult patients (two activations per day, achieving 0.4mm), the overload of the hardware (appliance and miniscrews) or of the involved anatomical structures can lead to unsatisfactory results [13–16]. In order to reduce these side effects, the rigidity of the expander used in this current study was improved and a novel 2-stage protocol was applied for miniscrew assisted palatal expansion (without Rapid expander activation) (MAPE) [17]. With this protocol (force-controlled polycyclic protocol: FCPC), an activation period is followed by a slow force-controlled polycyclic expansion period to weaken the circummaxillary sutures and enable maxillary expansion. This protocol has similarities with the Alt-RAMEC protocol [18] but is continued throughout the whole expansion period and and additionally combined with force control.

The purpose of this present study was to evaluate non-surgical maxillary expansion utilizing a miniscrew supported appliance with a novel 2-stage polycyclic expansion protocol in adult patients. The specific aims of the study were: (1) to evaluate the success rate of non-surgical maxillary expansion, (2) to measure the pattern of expansion in the midpalatal suture, and (3) to evaluate complications among the non-surgical patients.

## Materials and methods

This consecutive study was conformed to the Declaration of Helsinki and obtained the approval of the local Review Board (RB No. EK-2-2014/0016) and included a consecutive sample of adults who were treated between 2014 and 2016. Inclusion criteria for this study were: older than 18 years, and maxillary transverse deficiency greater than 2 mm measured by WALA ridge points at the lower first molars [19]. Exclusion criteria were: previous history of maxillary osteotomy, periodontal disease, previous orthodontic treatment, and dentofacial anomalies such as a cleft lip or palate.

**Fig. 1:**
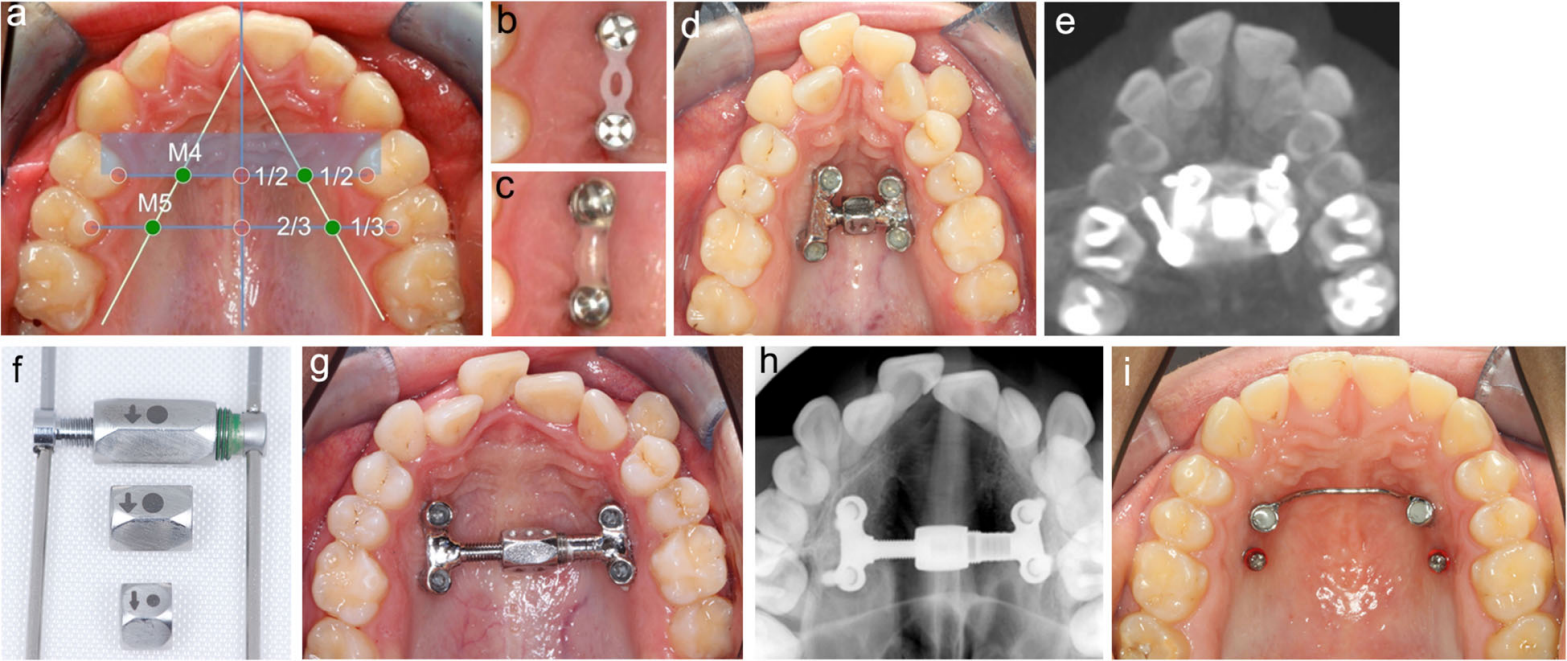
**a**-**k** Example of maxillary expansion with a MICRO-4 device in a 33.8 y **a** M4 and M5 positions for the orthodontic miniscrews **b** After placement, both screws on each side were connected with alastic chains. **c** This serves as a bridge to cover the screw heads with light curing resin to assure stability during 3 months of osseo-integration **d** MICRO-4 device with small hex nut **e** Initial occlusal x-ray **f** After reaching maximum opening of the jackscrew, the MICRO4 expander was removed and the small hex nut exchanged against a wider one **g** The same device was reinserted to continue the expansion procedure without the need of appliance reconstruction (in this case the right M5 orthodontic miniscrew needed to be relocated and the expander slightly adapted) **h** Final occlusal x-ray after expansion stop **i** Two year retention with bone-borne TPA. The orthodontic miniscrews in position M5 removed after insertion of TPA

This study comprised 33 patients (10 males, and 23 females) who were all treated by the same examiner (H.W.). All patients had to sign an informed consent. The average age (±SD) was 29.1 ± 10.2 years (min. 18.0 years, max. 58.0 years). All patients were treated with a MICRO-4 device that was used for treatment of transverse maxillary deficiency as previously described by Winsauer et al. [11]. Under local anesthesia, four orthodontic miniscrews (diameter, 2.5 mm; length, 14-16 mm; self-drilled type; Dual Top Jetscrew, Jeil Medical, South Korea) were inserted without surgical incision and without predrilling at the positions M4 (point on a line half way between palatal midline and palatal cusp of first premolar) and M5 (point on a line at the transition of the outer to middle third between palatal midline and palatal cusp of second premolar) in the anterior palate (without water-cooling and 25 rpm drill speed) and stabilized with temporary light-curing resin against each other [20]. After 12 weeks latency period (delayed loading for secondary stability), alginate impressions were taken from the upper jaw and the MICRO-4 device was fabricated in the laboratory and thereafter bonded to the screw heads (Phase II, Reliance Orthodontic, USA). In this study a novel 2-stage protocol (FCPC) was used: first, the device was activated for one week with a wrench turning the hex nut of the expansion screw two times per day by a one-sixth of a turn, achieving 0.34 mm per day (= activation period). Thereafter, the amount of force needed to activate the hex nut was measured by a spring scale (Push-Pull Spring Scale 10 N, Arbor Scientific, Ann Arbor, US) to assess the strain of the device (Fig. 1).

**Fig. 2:**
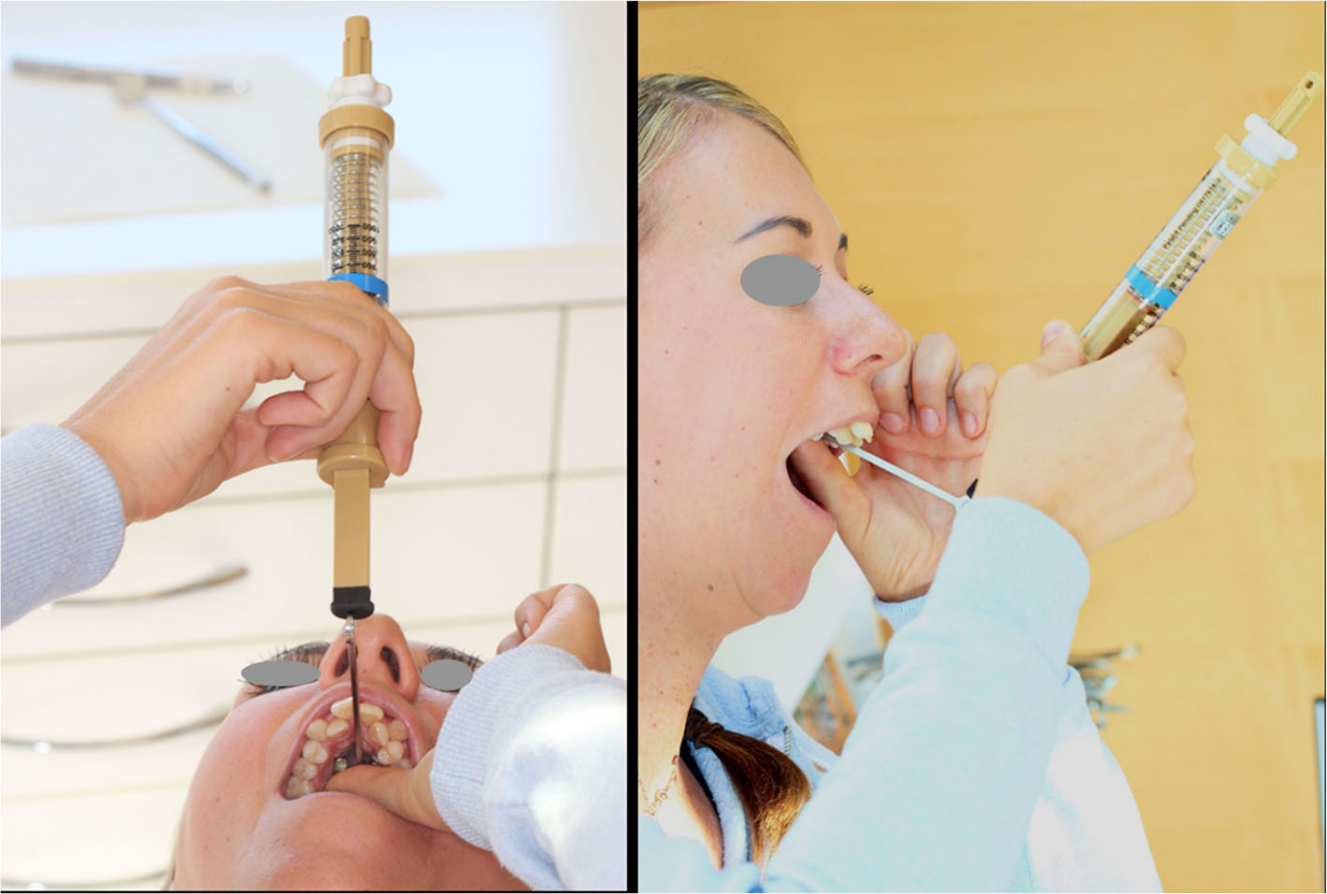
Force-controlled polycyclic expansion protocol (FCPC): force control by measuring the applied force at the end of the activating wrench. This is done by the patient twice a day with less than 500 cN turning power allowed

In order to weaken the circummaxillary sutures, all patients were then instructed to apply the following protocol twice per day: turn the hex nut 6 sides backward, and after 15 min, turn the hex nut forward 6 sides again. For each activation the force of the wrench was measured by the spring scale not exceeding 500cN. Every third day, the device was additionally activated by 0.17mm again not exceeding 500cN until the desired maxilla expansion was reached (= forced controlled polycyclic expansion period) (Fig. 2).

Success was defined when sufficient expansion of the midpalatal suture, according to the definition of transverse deficiency, was achieved without additional surgery (MAPE group). The patients without visible diastema within four months, were referred with the expansion device in place to the Department of Oral and Maxillofacial Surgery. SARPE was performed without osteotomy in the midpalatal bone under general anesthesia (SARPE group). Separation between the central incisors was accomplished using a scalpel blade no. 20 (Martin, Tuttlingen, Germany) as a chisel. After 5-days, the activation was started by turning the expansion screw at a rate of 0.5 mm per day (three turns a day) until the desired maxillary width was achieved. In both groups the MICRO-4 device was then left in place as a retention device for about 9 months and then replaced by a mini screw borne transpalatal arch for another 12-15 months.

A 43-year-old female patient was excluded from the study due to not following the activation protocol. The patient activated the expansion device more frequently, and with higher force. As a consequence, the right maxillary half, and the including nasal bone on one side expanded more than the contralateral side. Accordingly, the effective cohort study included 32 patients.

### Radiologic and Clinical Evaluation

**Fig. 3:**
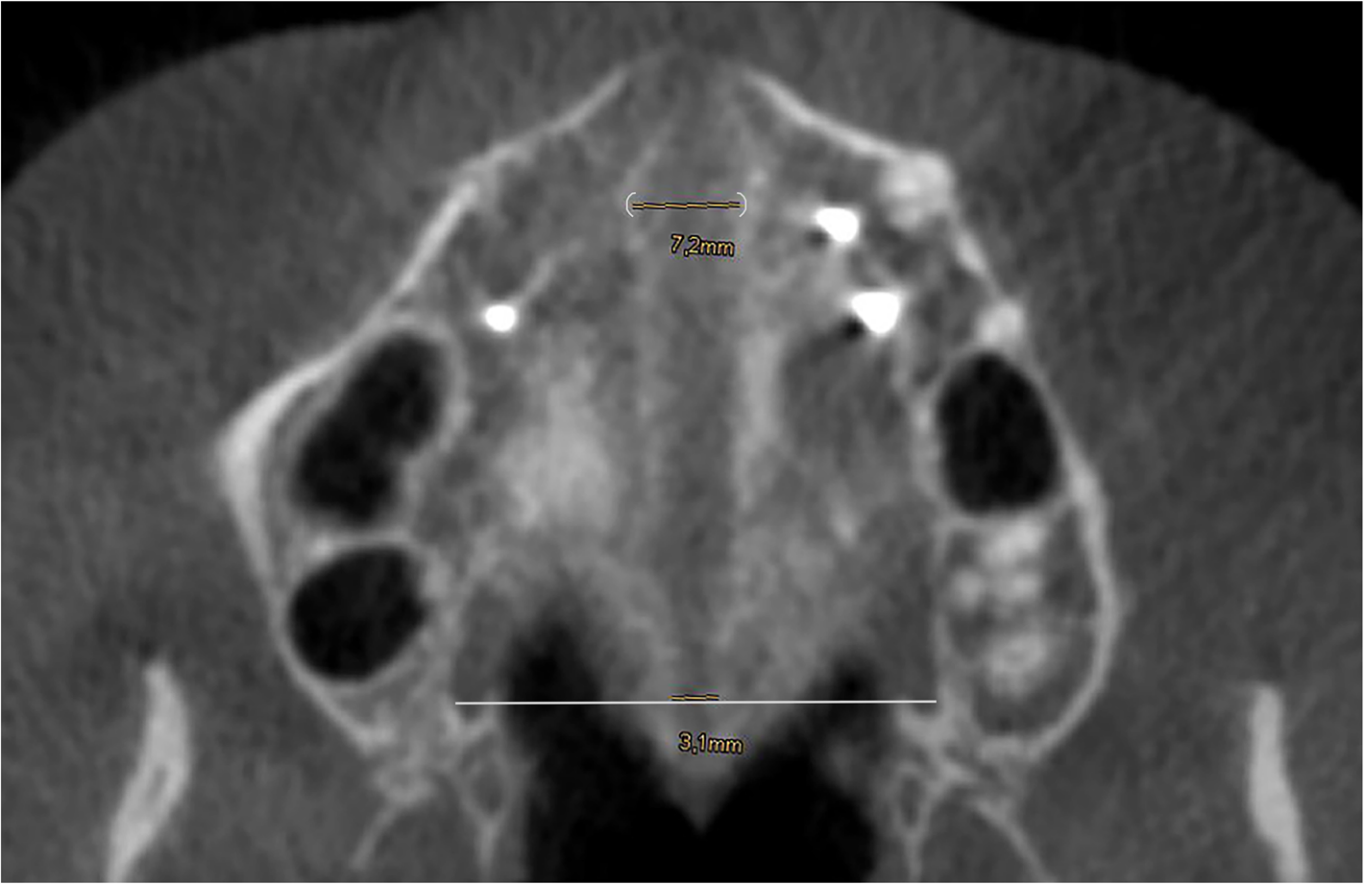
Measurements on CBCT after maximum of non-surgical expansion at the anterior and posterior palate. The nasopalatine foramen (white bracket) and the greater palatine foramina on both sides (white line) were references for measurements

A cone beam CT (KaVo 3D eXam, KaVo Dental GmbH, Biberach, Germany) was taken before treatment (range, 1–7 days) and after expansion (range, 2–4 months). The amount of widening of the midpalatal suture was measured at the level of the nasopalatine foramen (= anterior palate) and at the interconnection between the greater palatine foramina (= posterior palate) with the ruler tool of the CBCT software by one investigator (OP) (Fig. 3). All measurements were performed three times for each location and the average was calculated. The values were recorded and saved in an Excel spreadsheet (Microsoft Corp, Redmond, WA).

Complications were registered from the patient´s records. Dental (gingival irritation, increased periodontal probing depth, root resorption or damage, gingival recession, loss of vitality), tissue- (peri-implantitis, infection, ulceration) and hardware-related side effects (loosening or deformation of miniscrew or abutment, fracture or deformation of expansion screw), and anatomical complications (asymmetric expansion, fracture of bone) were recorded and saved in an Excel spreadsheet.

### Statistical Analysis

The statistical evaluations were carried out with the statistical program R (Version 3.1, R Foundation for Statistical Computing, Vienna, Austria). Normal distribution was tested with Shapiro-Wilk test and graphic data output. Continuous measures were represented by the means and standard deviations and the discrete features by absolute frequencies. To model the odds, for example in case of success, or complications, a generalized logistic regression with logit link function was used. Parameter significance of the generalized linear models are calculated using the Wald test, with the null hypothesis that the parameter is 0. A parameter is considered significant if p-value of the test is less than 0.05. Given the number of possible variables that can explain the outcome, stepwise regression to identify the best model was selected. Backward stepwise regression is used to find the best model starting from all possible independent factors. The models are assessed using the Bayesian Information Criteria (BIC), where higher BIC, indicates that the factor combination of the model is a better model. The final model is then used as the best fit model for the data we have. Stepwise regression is important to identify the right combination of variables that best fit the data we have, given the independent assumption of the variables. For significant testing between two distributions such as age, the non-parametric Wilcoxon test is used, to avoid operating under the normal distribution assumption in small datasets. For exact count significance tests like gender count, the fisher test was used. Power calculation of the study was done by simulating sampling from a statistical distribution representing the effect measured with the same sample size, while measuring the probability of having a significant outcome (< 0.05). The resulting power is then defined as the percentage of time we have obtained the significant result under the same sample size, and the uncertainty in the statistical distributions.

## Results

In 27 out of 32 patients the desired expansion was achieved without surgical intervention, resulting in a success rate of 84.4%. Using a multivariate binomial logistic stepwise regression of surgical intervention against age, gender, and duration of expansion the model showed significantly (p < 0.019 with power > 80%) high odds for the need of SARPE that increases by 17.9% per year above the baseline age of 18 years. For gender, this effect was not significant. Duration of expansion was eliminated as part of the stepwise regression. Further detailed descriptive data of the patients, amount, and pattern of expansion and are displayed in Table 1.

**Table 1:** Descriptive Data of the Treatment Groups and Intergroup Comparison

	MAPE group	SARPE group	*P*-Value
	*N* = 27 (84.4%)	*N* = 5 (15.6%)	
Age (years)	26.8 ± 8.2	41.3 ± 9.9	=0.005*
Age range (years)	18 - 49	31 - 58	
Age group (18-30) (n)	21	0	
Age group (30-40) (n)	4	3	
Age group (40-60) (n)	2	2	
Gender (Female/Male) (n)	19/8	3/2	=1
Expansion (anterior) (mm)	5.4 ± 1.5	6.3 ± 3.0	<0.0001*
Expansion (posterior) (mm)	2.5 ± 1.1	4.0 ± 2.1	=0.125
Screw expansion (mm)	6.4 ± 1.9	5.9 ± 0.9	=0.91
Duration of expansion (days)	81.2 ± 31.0	85.4 ± 75.5	=0.499
Retention (days)	298.9 ± 142.7	325.0 ± 230.5	=0.775

MAPE: successful non-surgical expansion using the MICRO-4 appliance; SARPE: failed non-surgical expansion using the MICRO-4 appliance; *Statistically significant for intergroup comparison (*P* < 0.05)

As demonstrated in Fig. 3, expansion of the midpalatal suture increased significantly both in the anterior and the posterior region of the palate. Among the MAPE group, the midpalatal suture opened in a V-shaped pattern in most patients (25/28), with the smaller increase observed in the posterior palate (Table 1). Expansion for the anterior and posterior palate was 5.41 ± 1.49 mm, 2.51 ± 1.07 mm, respectively. This difference was statistically significant (*p* < 0.001). A stepwise linear regression of the amount of expansion against duration, age, gender, and complication (as a binary indicator), showed that the duration of expansion is the only remaining significant (p = 0.031) factor that is correlated with the amount of expansion. On the other hand, neither age, gender, nor complications showed significant correlations.

Complications occurred in 18.5% of the subjects with successful non-surgical expansion using the technique described; the complications of the complete cohort are displayed in Table 2.

**Table 2:** List of complications for both groups (MAPE and SARPE)

Type of complication	MAPE group *N* = 27	SARPE group *N* = 5	Total *N* = 32
Soft tissue related	0	1	1 (3.1%)
Tooth related	0	0	0
Hardware related	5	1	6 (18.8%)
Patients with any complication	5/27 (18.5%)	2/5 (40.0%)	7/32 (21.9%)

MAPE: successful non-surgical expansion using the MICRO-4 appliance; SARPE: not successful non-surgical expansion using the MICRO-4 appliance

**Fig. 4:**
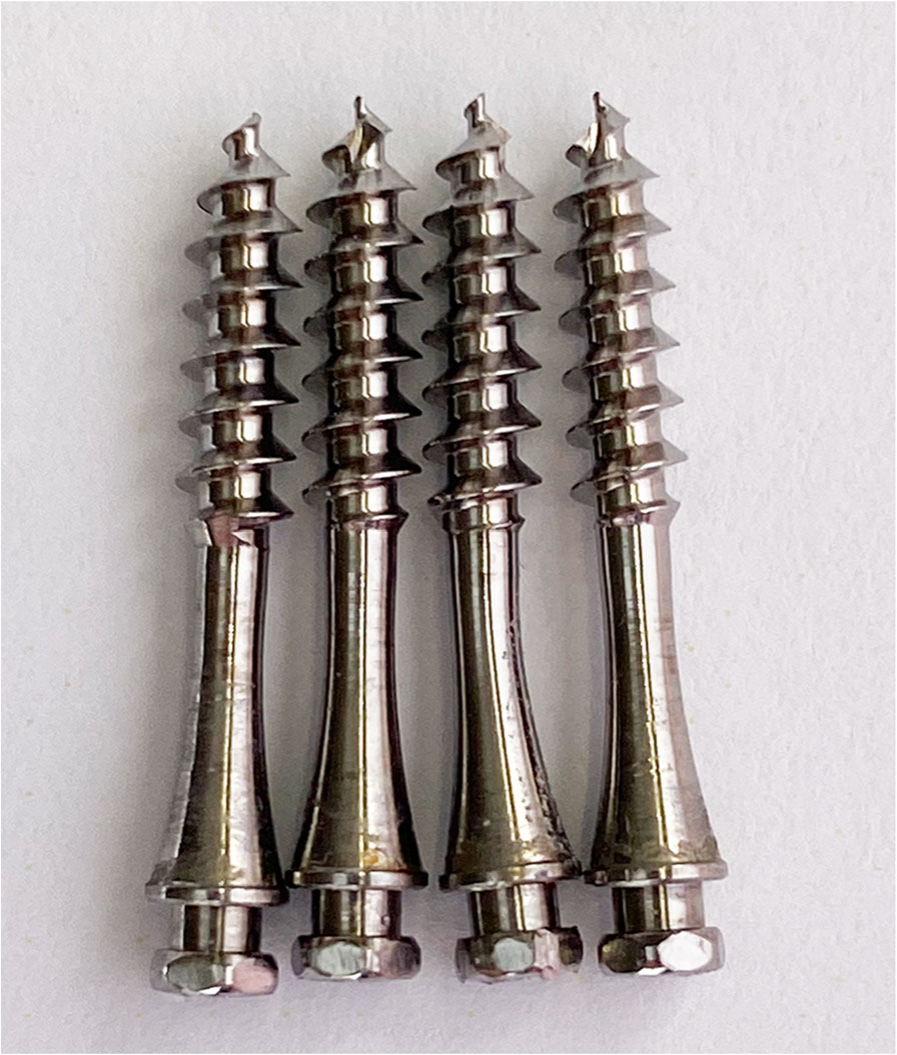
Minor deformation of mini screw as seen in 4 patients in the present study

Soft tissue inflammation (gingivitis or buccal tissue irritation) was observed in one patient of the SARPE group without clinical consequences. In six patients, hardware-related problems occurred during the expansion phase. In one patient, a loosening of the abutment was observed during the retention period, but this was without clinical relevance. In five further patients, a minor deformation of the mini-screw shaft (*n* = 4) or jack-screw (*n* = 2) was observed (Fig. 4).

Using a multivariate logistic stepwise regression model (complication or not, versus age and gender) showed that age has a significantly (*p* = 0.04, with power > 80%) high odd of 9.9%, for every extra year of age, above the baseline age of 18 years old. No significant effect was seen for gender.

## Discussion

The success rate of non-surgical expansion in adults using MAPE with the FCPC protocol was almost 84.4%, and is similar to those obtained in the recent literature [10, 21–23]. In contrast to the literature, the mean age of the patients in the present study was higher (29.1 years) than similar studies using MARPE for non-surgical expansion of mostly young adult patients (mean age from 17.1 to 23.3 years) [8, 10, 16, 24–27] or include subjects younger than 18 years old [8, 16, 24, 25, 28]. Moreover, successful expansion in the present study was achieved in patients up to 49 years. This evidence suggests that non-surgical palatal expansion, assisted by miniscrews or implants, is achievable even for older patients. This can be explained by various predictors for midpalatal suture expansion, such as patient´s individual anatomy, midpalatal suture maturation stage or density ratio [29], design, stability and location of the expansion appliance used, and the activation protocol applied for the expansion process [8, 21, 23, 27, 30]. In order to evaluate the success of non-surgical expansion, some studies have stated that the ossification of the midpalatal suture of each individual should be assessed by CBCT prior to treatment [29, 30]. The results of a recent study showed that the age differed significantly across midpalatal suture maturation stages, and correlated significantly with the midpalatal suture opening ratio [27]. Similarly, in the present study we found a significant association of age with both unsuccessful expansion and complications. This might be due to an increase of interdigitation occurring in the midpalatal and circummaxillary sutures in late adolescence, becoming more rigid as age progresses, mainly around 30 years of age [23]. A comparison of the results of this novel expansion technique with the staging classification method of Angelieri and coworkers will follow in a separate study including more subjects and to determine the intra- and interobserver reliability of midpalatal suture classification.

A relevant difference in our study that might explain the high success rate in older patients was the rigidity of the expander, the location of the inserted miniscrews, and the novel 2-stage protocol [17]. The MICRO-4 appliance was introduced by Winsauer et al. [11] which due to its rigidity can establish a more direct transfer of the expansion force to the hard palate. This aspect is especially interesting in older patients, since age-related changes of the suture may require more expansive force on the suture [17]. However, even with more rigid screws and abutments used some minor deformations were observed in our study as described in Table 2. Therefore, the needed force for expansion of a mature palate should never be underestimated.

Secondly, a further difference in our study was the location of the inserted miniscrews in the anterior palate. In general, placement of the miniscrews for the MARPE appliances is mostly in the middle or posterior part of the palate with less bone height, close to the midpalatal suture since these appliances are tooth-and-bone-borne with an additional fixation in the area of the first upper molar [10]. Because of the greater bone heights between 6 and 10 millimeters in the anterior part of the palate, this position is preferred for the fixation of the MICRO-4 device in our study [20]. The more stable anchorage of the miniscrews in this area may explain the high success rate. On the other hand, this location could also be the explanation of the anterior V-shaped expansion pattern in our study, resulting in 54% less expansion around the posterior palate as measured on the CBCT. Similar results with a V-shaped expansion of the dental arch in an antero-posterior plane were also reported with SARPE protocols when using bone-borne devices [9, 31]. However, the pterygomaxillary junction seems to be the point with the highest resistance as studied in a finite element study by Holberg et al. [3] and might explain the expansion pattern of the non-surgical cohort in the present study.

Thirdly, the second stage of the expansion protocol, the polycyclic closing and opening of the appliance, mimics oscillatory tensile and compressive strains, which are potent stimuli for modulating sutural growth by stimulating both osteogenesis and osteoclastogenesis [32]. Vij & Mao [33] stated that a cyclic loading protocol may have clinical implications as novel mechanical stimuli for modulating craniofacial growth in patients suffering from craniofacial anomalies and dentofacial deformities. This effect was used in our study and seems to weaken the circummaxillary sutures thus enabling successful expansion even in older patients. Based on the literature and the author´s experience, the protocol has been adapted in a pilot study to avoid technical or clinical complications such as loosening or deformation of screws and expander under rapid and continuous activation [34]. As mentioned above, a 43-year-old patient experienced an asymmetric expansion of the nasomaxillary complex with dislocation of the nasal bone. Possible factors that might lead to this complication were different bone density on both sides or asymmetric expansion force due to asymmetric screw position in the maxilla. Furthermore, this patient did not follow the polycyclic expansion protocol (way too high force applied) and as a consequence this subject was excluded in our study.

Usually, expansion protocols for MARPE (range 0.2 - 0.4 mm per day) [27] and for SARPE (0.5 - 1.0 mm per day) [7, 31] are much faster than the protocol used in our study. Therefore, the term “rapid” (as used in MARPE) is misleading and should not be used for non-surgical expansion in adult patients. However, some of the unsuccessful expansions observed in the present study were even younger than in the successful treatment group. The regression model showed an increase of almost 18% per year to experience unsuccessful expansion in our study. Although, a recommending baseline could not be found, this protocol can be used in all adult patients with the need for maxillary expansion. Due to the higher morbidity rate of SARPE procedure with the need for general anesthesia most patients prefer an orthodontic procedure rather than a surgical. Starting with MAPE utilizing the FCPC protocol, the SARPE procedure can be followed in case of missing diastema during the activation or early expansion period without any pre-treatment or changes in the hardware as described in our study. Interestingly, in the surgeon´s experience the bone between the central incisors was easy to separate using only a surgical blade as a chisel. The polycyclic activation seems to weaken the midline of the alveolar process and this positive side effect may reduce the risk of tooth damage even in cases with narrow space between the central incisors.

Expansion in adult patients might be associated with various complications and the prevalence of complications in patients undergoing SARPE is up to 34% [7]. Due to greater resistance in maturing maxillary bone, classical tooth-borne expanders cause a strong increase of dental side effects after attempting the expansion [35]. Some studies reported that bone-borne devices are associated with a risk of root lesions or infections, asymmetric maxillary expansion, periodontal damage, or loss of the distractor components [7, 31]. The complication rate of non-surgical expansion using MAPE was 18.5% in the present study. Although no severe complications of MARPE have been reported in the literature, the reduced elasticity of the bony structures in adults, might lead to microfractures with injury of nervous and vascular structures of the mid- or skull base [3]. The most frequent complication observed using MARPE were inflammation and hyperplasia of the mucosa around the miniimplant/screw or loosening or deformation of the screws used. A decrease in bone level and thickness at first molars was observed in 41% and undesirable effects like ulcerations, oedema of the palatal mucosa were observed in 22% of cases [15]. In a retrospective study on 69 patients, 5% of the miniscrews dislodged during expansion and 13% showed clinically visible mobility [16]. The most frequent cause for complications in our study was appliance-related. Generally, difficulties related to non-surgical expansion are associated with the device or with the expansion protocol itself, with the soft tissue around the anchorage of the device, the teeth, and the movement of the maxillary halves.

Using the regression model in our study the risk of complications increased by almost 10% per year above the baseline of 18 years. In other words, patients with 30 years had 1.2 folds higher risk for complication than compared with patients of 18 years old. However, problems with the appliance itself occurred also in two younger patients (23.5 and 25.6 years). Nevertheless, all appliance-related problems were without clinical relevance and a dislodge of miniscrews was not observed. The 12-weeks latency period after insertion of the miniscrews seems to allow osseointegration to enable sufficient force for maxillary expansion. As a consequence of the overload of the midface with asymmetric expansion in our study, all patients had to report the activation and progress with a written protocol daily during the first two weeks until the diastema was clearly visible.

Finally, the results of the present study are quite encouraging, showing that the protocol introduced, along with the MAPE appliance is forcing the expansion of the maxilla with an acceptable complication rate in adults. In case of unsuccessful expansion SARPE can be followed with the same appliance in place. The ease of surgery after pre-treatment justifies the protocol even in older patients.

### Limitations

Since this study had a small sample size it was not possible to evaluate different age groups (e.g. 20-30, 30-40, 40 and older) with respect to success and complications. Therefore, it is considered as a preliminary clinical study of lower evidence and in order to deal with the issue of small sample sizes, it would be more reliable to observe prospective cohort groups and to compare different activation protocols (gradual expansion versus forced controlled polycyclic expansion). A biased selection of subjects (i.e. gender, age, palatal vault) may have occurred in the course of clinical contingencies. The subjects were not randomly selected which limits the ability to generalize the results and could introduce bias. If CBCT is available for future studies a combination of midpalatal suture maturation staging or including density ratio as described by Angelieri et al. [29] should be implemented in the study design. However, further RCTs are needed to compare different activation protocols and to demonstrate how the patient´s age may influence treatment outcome in a larger number of patients and on long-term evaluation. Another question that raised during the present study is the influence of the pre-treatment on the amount of surgery during SARPE in cases of failed expansion. Is it still necessary to perform a complete osteotomy or can a minimal invasive surgery without splitting of the midpalatal suture, as demonstrated in our study, lead to the same results as a complete osteotomy and reduce morbidity of the patients?

## Conclusions

1. Successful non-surgical expansion using MAPE was observed in 84.4% adult subjects (25/28).
2. Midpalatal openings displays an anterior V-shaped pattern (22/25).
3. The complication rate for non-surgical expansion was 18.5% and age was the only relating factor studied.
4. A careful design and expansion protocol (polycyclic and slow) with MAPE seems beneficial to avoid unreliable results in older patients.

## Data Availability

The datasets used and/or analyzed during the current study are available from the corresponding author on reasonable request.

## Abbreviations

BIC: Bayesian Information Criteria
CBCT: Cone beam computed tomography
FCPC: Forced controlled polycyclic protocol
MAPE: Miniscrew assisted palatal expansion
MARPE: Miniscrew assisted rapid palatal expansion
MICRO-4: Mini-implant collar retained orthodontic expander on 4 miniscrews
RCT: Randomized clinical trial
RPE: Rapid palatal expansion
SARPE: Surgical assisted maxillary expansion
TPA: Transpalatal arch
WALA point: Point at the cross section of a central vertical line of the first lower molar crown and the band of keratinized soft tissue directly adjacent to the mucogingival line [19].
